# Estimating the completeness of death registration: An empirical method

**DOI:** 10.1371/journal.pone.0197047

**Published:** 2018-05-30

**Authors:** Tim Adair, Alan D. Lopez

**Affiliations:** Melbourne School of Population and Global Health, The University of Melbourne, Carlton, Victoria, Australia; Universidade Federal de Minas Gerais, BRAZIL

## Abstract

**Introduction:**

Many national and subnational governments need to routinely measure the completeness of death registration for monitoring and statistical purposes. Existing methods, such as death distribution and capture-recapture methods, have a number of limitations such as inaccuracy and complexity that prevent widespread application. This paper presents a novel empirical method to estimate completeness of death registration at the national and subnational level.

**Methods:**

Random-effects models to predict the logit of death registration completeness were developed from 2,451 country-years in 110 countries from 1970–2015 using the Global Burden of Disease 2015 database. Predictors include the registered crude death rate, under-five mortality rate, population age structure and under-five death registration completeness. Models were developed separately for males, females and both sexes.

**Findings:**

All variables are highly significant and reliably predict completeness of registration across a wide range of registered crude death rates (R-squared 0.85). Mean error is highest at medium levels of observed completeness. The models show quite close agreement between predicted and observed completeness for populations outside the dataset. There is high concordance with the Hybrid death distribution method in Brazilian states. Uncertainty in the under-five mortality rate, assessed using the dataset and in Colombian *departmentos*, has minimal impact on national level predicted completeness, but a larger effect at the subnational level.

**Conclusions:**

The method demonstrates sufficient flexibility to predict a wide range of completeness levels at a given registered crude death rate. The method can be applied utilising data readily available at the subnational level, and can be used to assess completeness of deaths reported from health facilities, censuses and surveys. Its utility is diminished where the adult mortality rate is unusually high for a given under-five mortality rate. The method overcomes the considerable limitations of existing methods and has considerable potential for widespread application by national and subnational governments.

## Introduction

Civil Registration and Vital Statistics (CRVS) systems should be the principal source of routine, timely and accurate data on deaths for national and subnational governments to draw on when planning health and social policy. However, decades of neglect of CRVS systems in many countries has meant that over half of global deaths are not registered [1]. Some of the reasons for incomplete vital registration data are the lack of a sound process of notification of vital events to the CRVS system, barriers to registration such as cost and lack of incentives, a poor legal framework to support registration, and a lack of coordination among multiple institutions to facilitate data transfer and compilation [1, 2]. In recent years, there have been significant investments made by national governments and bilateral, multilateral and philanthropic donors to rectify the poor state of much of the world’s CRVS systems. A major focus of this renaissance of interest in CRVS systems has been to ensure that all, or at least the vast majority, of deaths (and births) are notified to the CRVS system in countries so that planning can proceed with confidence, based on the knowledge that the vital registration data do not suffer from the biases inherent in incomplete data. As a consequence, governments at the national and subnational level need to routinely measure the completeness of death registration (defined as the number of registered deaths divided by the actual number of deaths in the population), not only to monitor performance of the CRVS system and target interventions accordingly, but also to know by how much to adjust death registration data to produce mortality statistics that will serve the current needs of planners.

Existing methods can be broadly classified into three groups:

- death distribution methods (DDMs), or indirect methods
- capture-recapture methods, or direct methods
- comparing registered deaths to total deaths estimated using a range of data sources and methods.

DDMs estimate the completeness of death registration data at ages 5 years and above by assessing the internal consistency of the age pattern of the population and the age pattern of deaths from the CRVS system, together with specific assumptions about the dynamics of the population. There are two main groups of indirect methods in use: Growth balance methods and Synthetic extinct generation methods [3]. Within each approach there is a method that assumes a stable population (ie a constant population growth rate and no migration) and requiring population data from one point in time, and a method that assumes a closed population and requiring population from two points in time to assess the completeness of intercensal death registration. An additional approach seeks to optimise the performance of both methods applied sequentially [4].

The two methods that assume a stable population, the Brass Growth Balance method and Preston–Coale method, are not widely used because this assumption simply does not apply in most present day populations due to rapid changes in birth and death rates, and hence the estimates are subject to significant uncertainty [5–7]. The Generalised Growth Balance and Bennett-Horiuchi methods are more widely used because the assumption of a population closed to migration is less restrictive than the assumption of a stable population, low levels of migration do not substantially impact completeness estimates, and any bias introduced by migration can be reduced by reducing the age range used to compute completeness ("age trims") [4, 8–10]. However the high rates of migration commonly found at the subnational level restricts their application to the national level. These methods also suffer from problems of timeliness because they only measure completeness of deaths between a country’s two most recent censuses, which can result in estimates applicable to a period well before the present day. As a result, countries that only have registered death data available for recent years may not be able to employ intercensal methods.

A common approach to applying DDMs is to use a Hybrid method, which involves using the relative completeness of the two censuses as measured by the Generalised Growth Balance method and applying this to the Bennett-Horiuchi method [11]. A study by Hill and others found the Hybrid method to be the most accurate DDM [12]. Another study by Murray and others suggested the most accurate DDMs are to use the Bennett–Horiuchi method for ages 55–80, the Generalised Growth Balance method for ages 40–70, and the Hybrid method for ages 50–70 [4]. However, uncertainty of completeness estimates from these approaches is considerable, with 95% uncertainty intervals measured to be approximately one-quarter of the estimate [4]. This is unlikely to be sufficiently narrow for monitoring improvements in death registration in populations. Further, DDMs assume the level of completeness is constant for all ages five years and over and that there is accurate reporting of age for both population and deaths [10]. Evaluation of DDMs supports their utilisation when all these assumptions, including no migration, are met [13]. However, the sensitivity of DDMs to violations of these assumptions can lead to incorrect estimates of death registration completeness, with the potential to seriously misinform efforts to improve vital registration systems.

Capture-recapture methods involve the matching of individual death registration data to another, theoretically independent, data source that reports deaths, such as a census, a survey or a health and demographic surveillance system (HDSS) site. These methods are a more time- and resource-intensive means to estimate registration completeness than DDMs. Where data are of good quality capture-recapture methods are more accurate because they are less reliant on the assumptions of indirect methods. Capture-recapture methods do, however, require that the data sources are independent of each other, have the same geographic boundaries and definition of residency, and can be successfully linked either by probabilistic or deterministic means. Completeness of registration is calculated for two sources using the straightforward Chardrasekar-Deming method [14]. More complex methods can also be employed where three sources are linked [15, 16]. Capture-recapture methods are particularly useful in that they can provide completeness estimates disaggregated by geography or age, although uncertainty can be considerable if death numbers are low [17]. Their main limitations are their time required, cost and complexity.

Finally, registration completeness can be estimated by dividing registered deaths by an estimate of total actual deaths, such as those made for countries by the Global Burden of Disease (GBD) Study and United Nations (UN) World Population Prospects [18, 19]. The GBD estimates total deaths by assessing under-five mortality and adult mortality separately, and then developing complete life tables [18]. Under-five mortality (*5q0*) is estimated from summary and complete births histories in censuses and surveys, as well as registration data, and annual estimates are generated using spatio-temporal Gaussian process regression that corrects for source-specific bias. Adult mortality (*45q15*) is estimated from registration, survey and census data. Completeness of registration data is assessed using three death distribution methods (Generalised Growth Balance, Bennett-Horiuchi and Hybrid), and a final estimate made by using spatio-temporal Gaussian process regression that combines these estimates with completeness of under-five registration. Estimates of adult mortality from survey and census data (adjusted sibling survival method or analysis of reported household deaths) are also made. Spatio-temporal Gaussian process regression is then made to estimate *45q15* over time using the completeness-adjusted registration, survey and census estimates, along with socio-economic and regional covariates. Complete life tables are generated from model life tables that use *5q0* and *45q15* estimates as inputs, along with a standard life table (a regional life table for countries with incomplete data, recent life tables for countries with good quality data). The UN World Population Prospects also estimates *45q15* using completeness-adjusted registration data for countries with incomplete registration data, and estimate full life tables by inputting *45q15* and *5q0* into model life tables [19]. For countries with complete registration data, the UN World Population Prospects data directly from life tables based on registered deaths, with the age pattern of mortality based on the Human Mortality Database [19].

Despite the importance of measuring the completeness of death registration, existing methods suffer from a number of problems. These are summarised in Table 1.

**Table 1:** Limitations of existing completeness methods.

Death distribution methods (indirect)	Capture-recapture methods (direct)	Comparing registered deaths to estimated total deaths
inaccuracy, where method assumptions are violated inconsistent estimates depending on the data and method used (when compared with other DDMs) rely on often unrealistic assumptions about population dynamics; including the assumption of the population being closed to migration which makes subnational application of the methods problematic lack of timeliness of estimates, especially for countries whose two most recent censuses were many years ago	time- and resource-intensive inaccuracy, where assumption of independence of data sources is violated complexity when linking three sources of data	considerable complexity that limits their application, especially at the subnational level

While the methodology of measuring under-five completeness by comparing risk of child death calculated from vital registration data with that calculated from (relatively reliable) censuses and surveys is reasonably well established, estimates of adult deaths required for the denominator are likely to have been based on one or more of the direct or indirect methods described above, with all their limitations [20]. One drawback of relying on the GBD and UN estimates can be inconsistent estimates for a specific country, even one with an established death registration system such as Peru, for example, where the UN estimates total deaths from 2010–15 of 855,000 versus the GBD’s estimate of 620,000 [18, 19]. The complexity of, and specialised analytical skills required, to implement and interpret these methods limits their use by national governments, and their intensive data requirements further limits their application at the subnational level.

In summary, the current suite of methods available to estimate the completeness of registration is confusing, cumbersome and unsuitable to their widespread application by national and subnational governments. There is a clear need for a relatively simple and accurate method to estimate completeness of registration utilising data that are timely and widely available, including at the subnational level, that avoids these disadvantages but which preserves the expected relationships among the principal determinants of mortality levels in a population. Such a method should be able to be implemented by national and subnational governments with minimal training. This paper presents a novel method to estimate completeness of registration that seeks to meet these objectives, based on modelling the key drivers of the crude death rate in populations.

## Methods

### Predicting completeness of death registration

The crude death rate (CDR) is a summary measure of mortality that essentially reflects the level and age pattern of mortality in a population, and the population’s age structure. CDR is defined as follows:

$$CDR=\frac{Total\;deaths}{Population}*1000=(\frac{\sum_{x}m_{x}*P_{x}}{\sum_{x}P_{x}})*1000$$

where *m*_*x*_ is the age-specific death rate at age *x* and *P*_*x*_ is population at age *x*.

The risk of mortality is highest in infants and young children, and then again among older adults, and especially at the oldest ages. Hence, it is likely that the CDR will be higher in ageing populations where there are many more people alive at older ages where the risk of death is highest. This is also true, but to a lesser extent, for populations with very high birth rates, and hence a large fraction of young people aged below five years who may experience a high risk of death.

In all populations with a death registration system, national or subnational, the registered crude death rate (*RegCDR*) should be readily available. We define:

$$RegCDR=\frac{Registered\;deaths}{Population}*1000$$

It follows that completeness of death registration is the *RegCDR* divided by the product of age-specific death rates and population.

$$Completeness=\frac{Registered\;deaths}{Total\;deaths}=\frac{RegCDR}{CDR}=\frac{RegCDR}{(\frac{\sum_{x}m_{x}*P_{x}}{\sum_{x}P_{x}})*1000}$$

As this equation demonstrates, completeness of death registration has a positive relationship with *RegCDR*, a negative relationship with the level of mortality and a negative relationship with an older population age structure. We use this equation to model the relationship between completeness of death registration, *RegCDR*, population age structure and the risk of child mortality.

Data from 110 countries since 1970 were utilised; these data are based on plausible estimates of completeness prepared for the GBD Study using the methods described earlier [18]. For each country-year included in the dataset, the registered and estimated CDR were extracted and modelled.

**Fig 1:**
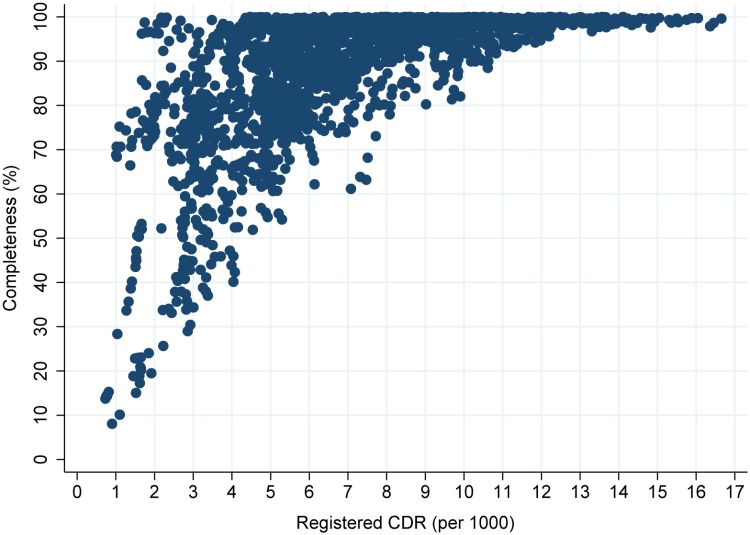
Death registration completeness by registered CDR, 110 countries, 1970–2015.

Completeness and *RegCDR* demonstrate a curvilinear relationship, which shifts according to the level of mortality and population age structure (Fig 1). At lower levels of *RegCDR*, completeness varies considerably; countries with relatively low levels of mortality and young populations can have completeness close to 100% (e.g. some Gulf states), while those with higher mortality and/or older populations can completeness less than 30%. At higher levels of *RegCDR*, variation in completeness is lower; in no country with a *RegCDR* of 9/1000 or above is completeness less than 80%. Some countries, such as Japan, have a very high CDR despite low mortality because of a very old age structure.

### Model

**Fig 2:**
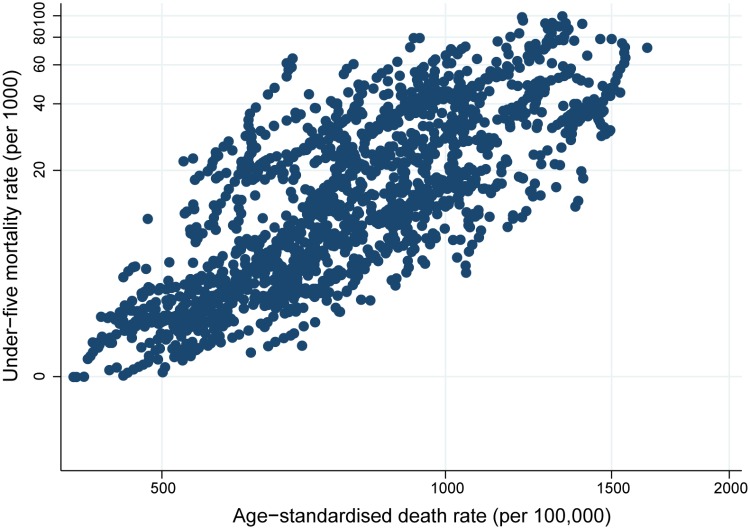
Under-five mortality rate by age-standardised death rate (both log scale), 108 countries, 1990–2015.

The model to predict completeness is based on the relationship between *RegCDR*, the true level of mortality and population age structure. Data on the *RegCDR* and population age structure are readily available at the national and subnational level. Measurement of the true level of mortality is more problematic. In populations without complete death registration systems, reliable measures of child mortality are widely available from multiple sources (surveys and censuses) where complete and summary birth history methods utilise retrospective reporting of births by mothers [21]. Measurement of adult mortality (*45q15*) is more problematic due to less robust methods, difficulties in identifying the most appropriate respondent to report these deaths and scarcer availability of data, especially at the subnational level in countries with incomplete CRVS systems. Therefore, we included the under-five mortality rate (*5q0*) in the model as a proxy measure of overall mortality levels in a population. This measure of the overall level of mortality may not be optimal in countries where the level of adult mortality is higher or lower than expected, given the level of *5q0*, thus reducing the predictive power of the model. Such countries would include those with high mortality from HIV/AIDS, and/or where there are wars or other exceptional mortality ‘shocks’, including alcohol abuse. We have excluded these country-years from the dataset. However, there is a strong relationship between *5q0* and the overall level of mortality, measured by the age-standardised death rate, for countries in our dataset (Fig 2). Further, the software program ANACONDA, which assesses vital statistics data quality, includes estimates of *5q0* as well as other data inputs to enable estimation of the completeness of registration [22].

The model makes use of the wide availability of data on *5q0* by also including a measure of the completeness of under-five death registration, calculated as the *5q0* from registration data divided by the estimated actual level of *5q0*. We included this variable because it is expected to be strongly associated with registration completeness over all ages. This variable may not be as suitable to apply to registration data derived largely from deaths that occur in health facilities, because deaths of young children are more likely to occur in health facilities than deaths at older ages. Completeness of under-five death registration would therefore be relatively high compared to completeness of deaths at all ages, and so predicted completeness may be too high. To allow for this possible effect, an additional model was developed (Model 2) that does not include under-five registration completeness. A variable to reflect the calendar year of the data was also included.

The models are as follows:

$$logit\;(C_{jk}^{All})=\beta_{0}+{{RegCDRsq}_{jk}*\beta }_{1}+{{RegCDR}_{jk}*\beta }_{2}+{\%65}_{jk}*\beta_{3}+{ln(5q0)}_{jk}*\beta_{4}+C_{jk}^{5q0}*\beta_{5\;}+k*\beta_{6\;}+e_{jk}+\gamma_{j}$$ (1)

$$logit\;(C_{jk}^{All})=\beta_{0}+{{RegCDRsq}_{jk}*\beta }_{1}+{{RegCDR}_{jk}*\beta }_{2}+{\%65}_{jk}*\beta_{3}+{ln(5q0)}_{jk}*\beta_{4}+k*\beta_{5\;}+e_{jk}+\gamma_{j}$$ (2)

where $C_{jk}^{All}$ is the completeness of registration at all ages, *logit($C_{jk}^{All}$)* is $ln(\frac{C_{jk}^{All}}{1-C_{jk}^{All}})$, *RegCDR*_*jk*_ is the registered CDR, *RegCDRsq*_*jk*_ is the square of *RegCDR*, %65_*jk*_ is the fraction of the population aged 65 years and over, *ln*(5*q*0)_*jk*_ is the natural log of the under-five mortality rate, $C_{jk}^{5q0}$ is the completeness of the registered *5q0*, *k* is calendar year, *e* is an error term, *γ* is a country-level random effect, *j* is country and *β*_0_ to *β*_6_ are the coefficients. Predicted completeness is converted using the inverse logit: $\frac{e^{logit(C_{jk}^{All})}}{e^{logit(C_{jk}^{All})}+1}$.

A squared term for *RegCDR* was included because it more accurately predicts completeness at lower levels of *RegCDR* than either *RegCDR* alone or the natural log of *RegCDR*. The percentage of the population aged 65 and over is intended to capture the impact of population age structure; inclusion of the dependency ratio (population aged 65 years and over divided by the population aged 15–64 years) produced near identical results. As mentioned, a separate model (Model 2) was included without using under-five mortality completeness. The two models were also developed separately for males and females to enable separate prediction of male and female completeness. The sex-specific models include sex-specific data for every variable except completeness of under-five death registration which is likely to be similar between males and females [21]. An additional model to describe the level of mortality in adulthood, the risk of adult mortality (*45q15*), was found to have little effect on the predictive performance of the models (R-squared increased from 0.851 to 0.863 for model 1, both sexes). Completeness estimated from Model 1 can be converted to completeness for ages five years and over using this formula:

$$C_{jk}^{5+}=\;\frac{{RegDeaths}_{jk}^{5+}}{\left(\frac{{RegDeaths}_{jk}^{All}}{C_{jk}^{All}})-(\frac{{RegDeaths}_{jk}^{0-4}}{C_{jk}^{5q0}}\right)}$$

where $C_{jk}^{5+}$ is completeness at ages five years and over, ${RegDeaths}_{jk}^{5+}$ is registered deaths at ages five years and over, ${RegDeaths}_{jk}^{All}$ is registered deaths at all ages and ${RegDeaths}_{jk}^{0-4}$ is registered deaths at ages less than five years.

### Data

The model utilises mortality and population data from the GBD 2015 database as the observed or ‘true’ level of mortality in a population, against which to measure the predictive performance of the model [18]. The methods employed by the GBD are described in detail above [18]. We calculated completeness of death registration by dividing registered deaths by estimated of total deaths in the population.

A total of 2,451 country-years from 110 countries between 1970 and 2015 were included to construct the model. From the original data, some country-years were deleted, as mentioned above. These are country-years with one or more of the following characteristics:

- estimated completeness of over 100%,
- less than 1,500 estimated total deaths (to minimise the impact of stochasticity),
- implausible or inconsistent completeness of under-five mortality registration, where either there is at least 50 percentage points difference compared with completeness at all ages, or completeness at ages five years and over is calculated as greater than 100%
- excessively high adult mortality compared with under-five mortality due to the HIV/AIDS pandemic (Botswana, Zimbabwe, Lesotho), high numbers of conflict deaths (Iraq 1987–88, Cyprus 1974 and Israel 1973), natural disasters (Honduras 1974, Armenia 1988, Venezuela 1999, Sri Lanka 2004) and high numbers of alcohol-related deaths (Russia 1994–96), or
- implausibly high levels of observed completeness based on additional data available for some of the countries (Colombia between 1998–2015, and Ecuador).

Earlier country-years that were clear outliers (i.e. predicted completeness far different from observed completeness) were also deleted (Peru 1970–73, Tunisia and Lebanon, Guatemala 1970–76, Madagascar 1970–72, Egypt 1970–79, Maldives 1975–78, Mongolia 1970, Bolivia 1976–77, Suriname 1972, El Salvador 1970).

A full list of countries in the dataset is shown in S1 Table.

All models were developed using Stata 13 [23].

### Measures of fit and validity

The goodness of fit of the model was tested using a range of methods. The overall degree of fit of the model to the data (R-squared) was calculated using the following formula, excluding country-level random effects:

$$R-squared=1-\frac{\sum_{j}\sum_{k}{\left(C_{jk}^{All}-{\hat{C}}_{jk}^{All}\right)}^{2}}{\sum_{j}\sum_{k}{\left(C_{jk}^{All}-{\overset{-}{C}}_{jk}^{All}\right)}^{2}}$$

where ${\hat{C}}_{jk}^{All}$ is predicted completeness. ${\overset{-}{C}}_{jk}^{All}$ is mean observed completeness.

The mean absolute error (MAE) was calculated to assess any systematic bias in over- or under-predicting completeness, while the root mean squared error (RMSE) was calculated to assess the overall level of prediction error. The MAE and RMSE were assessed at various levels of observed completeness to indicate how the models will perform across a range of populations.

Out-of-sample validation of the model was also conducted at both the country-year and country level. The models were first estimated using 80% of country-years/countries available, and the results were used to predict completeness in the remaining 20% of country-years/countries. This process was repeated five times so that all country-years/countries were included in the out-of-sample component. All out-of-sample predictions were combined, and the MAE and RMSE of these combined predictions compared with those of the model developed using the full dataset.

The sensitivity of the models to uncertainty in *5q0* was tested by varying *5q0* for each country-year by one standard error, and comparing predicted completeness with that where the original *5q0* was used. We tested sensitivity for country-years where true completeness is less than 90%, because countries with high completeness are likely to have lower uncertainty in estimated *5q0*.

### Application to national and subnational data

We applied the models to national and subnational data not included in the dataset used to develop the model. Firstly, we estimated completeness for eight countries and two cities for which we had access to vital statistics through collaboration in the Bloomberg Data for Health Initiative that were not publically available in international databases. We compared these estimates of completeness with observed completeness calculated using the GBD estimate of total deaths.

To demonstrate the utility and ease of application of the model to estimate levels of subnational death registration completeness, we used the models to estimate registration completeness for Colombia’s *departmentos* and capital district in 2014 using publically available data [24, 25]. The *5q0* was estimated as the average of *5q0* from the 2010 and 2015 Demographic and Health Surveys, scaled to the GBD estimate of *5q0* for Colombia for 2014 [26, 27]. To further assess sensitivity of the model to uncertainty in *5q0*, predicted completeness was calculated using the input *5q0* plus or minus one standard error. We also compared estimates of registration completeness at ages 5+ in Brazilian states in 2000–2010 with those made by Queiroz and others, who used both the Generalised Growth Balance method and the Hybrid method described above [28]. Data from Brazil to apply the empirical method included publically available data from the Mortality Information System (SIM), and *5q0* data estimated by the GBD [29–31]. As noted earlier, established methods are likely to be much more difficult to apply, and interpret, at the subnational level.

## Results

Results from each model are presented for males and females, and for both sexes (Table 2). All predictor variables are highly significant. The direction of each coefficient is as expected; both the under-five mortality rate and percentage of the population over the age of 65 have a negative relationship with completeness (because they increase the CDR). The R-squared for both sexes is 0.851 for Model 1 and 0.811 for Model 2. The stronger performance of Model 1 is to be expected given that it includes the under-five registration completeness variable, which is likely to contain significant information content for predicting overall completeness as confirmed by the positive coefficient. The R-squared for both models is very similar for males and both sexes combined, but is slightly lower for females. The MAE indicates that each model slightly over-predicts completeness.

**Table 2:** Results from models of death registration completeness, both sexes, males and females.

**Model 1**	**Both sexes**			**Male**			**Female**		
	**Coef.**	**z**	**P>z**	**Coef.**	**z**	**P>z**	**Coef.**	**z**	**P>z**
RegCDR squared	-0.0177	-6.51	0.000	-0.0174	-7.79	0.000	-0.0198	-6.30	0.000
RegCDR	0.6375	12.10	0.000	0.5957	12.87	0.000	0.6959	12.76	0.000
%65+	-13.8914	-8.52	0.000	-12.9528	-8.11	0.000	-17.4154	-11.68	0.000
ln(*5q0*)	-1.1136	-13.60	0.000	-1.1266	-14.15	0.000	-1.1720	-14.59	0.000
C_*5q0*_	2.2063	9.71	0.000	2.0030	9.31	0.000	1.9387	8.81	0.000
Year	-0.0174	-5.86	0.000	-0.0188	-6.27	0.000	-0.0144	-4.92	0.000
Constant	29.3677	5.08	0.000	32.3442	5.52	0.000	23.5542	4.15	0.000
N	2,451			2,263			2,319		
R-squared	0.851			0.850			0.828		
MAE	0.6			0.5			0.7		
RMSE	2.1			2.2			2.3		
**Model 2**	**Both sexes**			**Male**			**Female**		
	**Coef.**	**z**	**P>z**	**Coef.**	**z**	**P>z**	**Coef.**	**z**	**P>z**
RegCDR squared	-0.0238	-8.60	0.000	-0.0227	-10.06	0.000	-0.0255	-8.02	0.000
RegCDR	0.8419	16.50	0.000	0.7620	16.96	0.000	0.8841	17.02	0.000
%65+	-19.6118	-12.02	0.000	-17.3543	-10.79	0.000	-22.1099	-15.00	0.000
ln(*5q0*)	-1.5135	-19.68	0.000	-1.4798	-19.75	0.000	-1.5262	-20.26	0.000
Year	-0.0251	-8.07	0.000	-0.0265	-8.58	0.000	-0.0217	-7.17	0.000
Constant	44.3755	7.35	0.000	47.3778	7.89	0.000	37.7887	6.46	0.000
N	2,451			2,263			2,319		
R-squared	0.811			0.816			0.783		
MAE	0.7			0.6			0.7		
RMSE	2.1			2.2			2.2		

Coef.: Coefficient. N: Number. MAE: mean absolute error. RMSE: Root mean squared error. Figures shown for MAE and RMSE are percentage points. There are a lower number of country-years in the male and female models because some were removed according to the criteria described above. Random effects are presented in S2, S3, S4, S5, S6 and S7 Tables.

**Fig 3:**
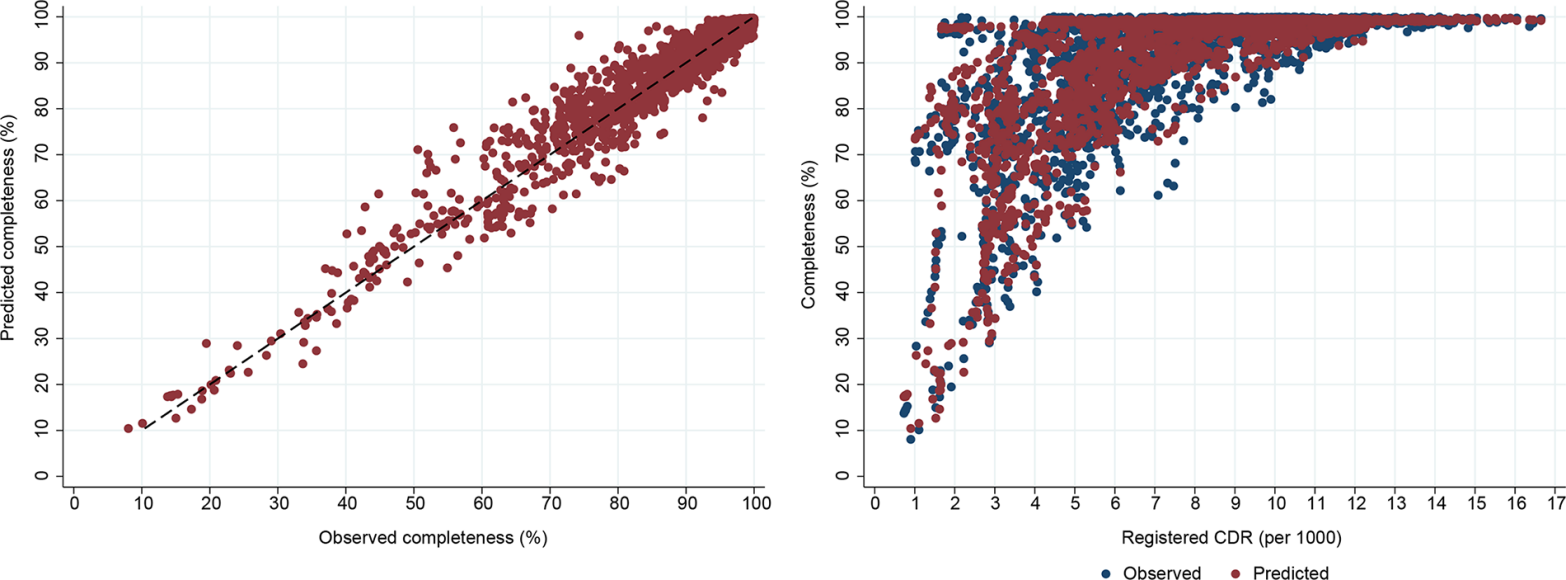
Predicted versus observed death registration completeness, and predicted versus observed death registration completeness by registered CDR, Model 1, both sexes.

**Fig 4:**
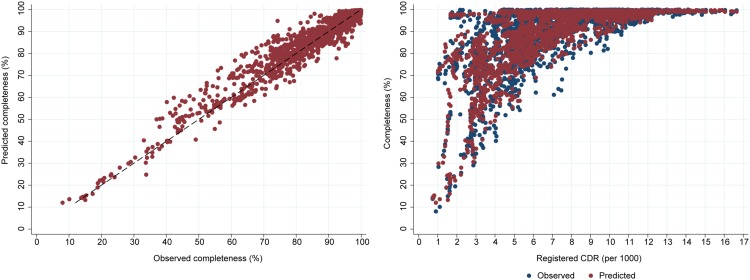
Predicted versus observed death registration completeness, and predicted versus observed death registration completeness by registered CDR, Model 2, both sexes.

Scatter plots of predicted versus observed completeness confirm the high predictive accuracy of the model (Figs 3 and 4). For both models, they demonstrate a slight over-estimate of completeness around a true completeness level of 40–60%. Encouragingly, the models are able to reliably predict completeness of registration across a wide range of levels of *RegCDR*. For example, at *RegCDR* of less than 3/1000, predicted completeness ranges from close to 100% for some countries (i.e. Gulf countries with low mortality and young age structures) to much lower levels of completeness due to poorly performing vital registration systems, matching the range of observed completeness. For a handful of country-years where the registered CDR is above 7/1000 and completeness is less than 80%, the models perform less well, but adequately for their intended policy purposes. These are predominantly country-years from the 1970s and 1980s, where the model over-estimates completeness by a median 10 percentage points.

The high degree of predictive accuracy at the highest levels of completeness is evidenced by the low MAE, RMSE and narrow 95% confidence intervals (Table 3). At levels of observed completeness between 30% and 60%, MAE and RMSE are higher (MAE showing a slight over-estimation of completeness and RMSE an average error of 5 percentage points), and 95% confidence intervals are wider (approximately 10 percentage points). The models are also sufficiently accurate for policy purposes at observed completeness levels below 30%, despite such country-years comprising a low proportion of the data.

**Table 3:** Model goodness of fit by level of observed death registration completeness (%), both sexes.

Observed completeness (%)	Model 1					Model 2				
	MAE	RMSE	Mean pred. compl.	Mean lower 95% CI	Mean upper 95% CI	MAE	RMSE	Mean pred. compl.	Mean lower 95% CI	Mean upper 95% CI
90<100	0.2	1.1	97.7	97.2	98.0	0.0	1.2	97.6	97.1	98.0
80<90	0.7	3.5	85.8	83.5	87.9	0.9	3.0	86.0	83.4	88.2
60<80	2.1	5.2	74.2	70.5	77.7	2.6	4.7	74.7	70.7	78.4
30<60	2.8	4.8	50.4	45.1	55.7	4.7	5.8	52.2	46.5	57.9
<30	1.0	2.3	19.7	15.5	24.7	1.1	1.4	19.9	15.4	25.3

MAE: mean absolute error. RMSE: root mean squared error. Pred. compl.: Predicted completeness. 95% CI: 95% confidence interval. Figure shown for MAE and RMSE are percentage points.

The out-of-sample validation test shows quite similar RMSE and MAE to the full sample at the country-year level (Table 4). As expected, the models do not perform as well at the country-level largely because there are no country-level random effects for the out-of-sample countries.

**Table 4:** Model goodness of fit by level of observed death registration completeness (%), full sample and country-year and country level out-of-sample validation, Models 1 and 2, both sexes.

Observed compl.	Model 1			Model 2		
	Full sample	Country-year level	Country level	Full sample	Country-year level	Country level
RMSE	2.1	2.3	3.5	2.1	2.2	3.8
MAE	0.6	0.6	1.0	0.7	0.8	2.0

Compl.: Completeness MAE: mean absolute error. RMSE: root mean squared error. Figures shown are percentage points.

For Model 1, the median change in completeness from a one standard error increase in *5q0* is -2.2 percentage points, the mean change is -2.9 percentage points, and for 90% of country-years the change is between -5.7 and -0.5 percentage points (only using country-years with true completeness of less than 90%). Model 2 is less sensitive to changes in *5q0*; the median change in completeness from a one standard error increase in *5q0* is -1.5 percentage points, the mean change is -1.9 percentage points, and for 90% of country-years the change is between -3.5 and -0.3 percentage points. The greater sensitivity of Model 1 is because it includes two variables (*5q0* and *5q0* completeness) impacted by uncertainty in *5q0*.

Application of the model to predict completeness for eight countries and two cities in the Bloomberg Data for Health Initiative not included in the dataset used to build the models, but with a very wide range of true completeness, shows quite close agreement between predicted and observed (i.e. GBD estimated) completeness (Table 5). Models 1 and 2 predict similar levels of completeness, with the largest difference being eight percentage points. For Model 1, the largest absolute discrepancy from observed completeness is 6 percentage points (53% versus 59%). Application of Model 2 to this country, which is solely based on health facility data, results in a more accurate prediction. Overall, Model 2 is slightly less accurate, with the biggest discrepancy being a predicted completeness of 15% for the country with observed completeness of 5%.

**Table 5:** Predicted and observed death registration completeness (%), eight countries and two cities in Data for Health Initiative, Models 1 and 2, both sexes.

Country/city	Predicted completeness		Observed completeness
	Model 1	Model 2	
Country 1	80	84	81
Country 2*	53	55	59
Country 3	47	54	47
Country 4	23	32	28
Country 5	8	13	12
Country 6	9	15	5
Country 7	6	8	5
Country 8	5	7	3
City 1	98	97	100
City 2	99	99	100

Observed completeness for other countries is based on estimated total deaths in the GBD. Specific countries and cities are unable to be identified under country collaboration agreements governing the Data for Health Initiative. The 20 Data for Health Initiative countries/cities are Bangladesh, Brazil, Colombia, Ecuador, Ghana, Indonesia, Malawi, Morocco, Mumbai, Myanmar, Papua New Guinea, Peru, Philippines, Rwanda, Shanghai, Solomon Islands, Sri Lanka, Tanzania, Turkey and Zambia. Countries in table are ordered by level of observed completeness.

* Data from health facilities only, observed completeness based on UN World Population Prospects (UN 2017).

Predicted completeness for *departmentos* in Colombia exhibits substantial variation, ranging from 96% in Caldas and Meta (Model 1) to 25% in La Guajira (Model 1) (Table 6). Predicted completeness according to the two models is within 10 percentage points, with the exception of Putumayo, where the completeness of under-five mortality registration is low (33%). For most *departmentos*, a one standard error change in *5q0* changes predicted completeness a few percentage points in each direction; the average change for Model 1 is ± 5 percentage points (maximum of 10) and for Model 2 is ± 3 percentage points (maximum of 6).

**Table 6:** Predicted completeness and input data by *departmento* of residence (%), Colombia, 2014, Models 1 and 2, both sexes.

*Departmento*	Predicted completeness (%)		Reg CDR	% 65+	*5q0*	*5q0* compl. (%)
	Model 1 (± 1 *5q0* S.E.)	Model 2 (± 1 *5q0* S.E.)				
National	84 (83–86)	87 (86–87)	4.4	7.3	17	63
Antioquia	88 (84–92)	90 (88–92)	4.5	7.6	14	64
Arauca	90 (84–96)	94 (91–96)	4.1	5.0	12	61
Atlántico	91 (86–94)	88 (85–90)	4.5	6.8	18	85
Bogotá	83 (78–89)	84 (81–87)	3.8	7.2	15	66
Bolívar	80 (72–87)	79 (74–83)	3.6	6.8	18	67
Boyacá	85 (78–92)	89 (85–92)	5.0	9.6	14	57
Caldas	96 (92–98)	96 (94–98)	5.8	9.4	9	83
Caquetá	82 (75–89)	85 (81–89)	3.6	5.4	16	59
Casanare	94 (88–96)	92 (89–95)	3.5	4.7	11	88
Cauca	74 (67–82)	78 (73–82)	3.8	7.5	19	54
Cesar	79 (72–86)	79 (75–83)	3.8	5.5	24	66
Chocó	53 (46–61)	53 (49–59)	2.7	4.7	34	51
Córdoba	82 (72–91)	81 (76–87)	3.9	6.4	19	67
Cundinamarca	92 (86–96)	92 (89–95)	4.4	7.5	11	74
Huila	81 (73–89)	85 (81–90)	4.5	6.7	20	56
La Guajira	25 (21–29)	29 (26–33)	2.0	5.0	45	28
Magdalena	85 (79–91)	85 (81–88)	4.1	6.3	18	71
Meta	96 (93–97)	95 (93–96)	4.6	6.0	11	100
Nariño	72 (65–80)	79 (75–84)	3.8	7.4	18	47
Norte de Santander	87 (82–92)	91 (89–93)	5.1	6.9	17	56
Putumayo	55 (47–64)	68 (62–74)	3.1	5.0	27	33
Quindío	89 (85–93)	94 (92–96)	6.4	9.1	16	48
Risaralda	93 (89–97)	94 (92–96)	5.7	8.8	13	76
Santander	90 (85–95)	91 (88–93)	5.0	8.2	14	71
Sucre-Archipiélago de San Andrés y Providencia	91 (86–93)	86 (83–89)	3.8	7.1	14	93
Tolima	84 (79–90)	89 (86–91)	5.6	9.0	19	55
Valle del Cauca	91 (88–94)	94 (92–95)	5.3	8.1	13	64
Vaupes-Guaviare-Amazonas-Vichada-Guainia	72 (67–77)	70 (67–73)	2.4	4.1	20	65

S.E.: Standard error. Compl.: completeness. Archipiélago de San Andrés y Providencia is combined with Sucre because the population of the former is less than 100,000. Vaupes, Guaviare, Amazonas, Vichada and Guainia are combined because each population is less than 110,000.

Analysis of Brazilian states shows there is high concordance between Model 1 with the Hybrid method (Generalised Growth Balance Method to adjust census completeness, and SEG method to estimate completeness), showing a root mean squared difference of three percentage points and mean absolute error of plus three percentage points (Table 7). There was, however, less concordance between Model 1 and the Generalised Growth Balance Method (root mean squared difference of six percentage points and mean absolute error of minus five percentage points). The largest difference in the comparison of Model 1 and the Hybrid method is 14 percentage points in the state of Para.

**Table 7:** Predicted completeness and Queiroz et al (2017) estimates of completeness by state of residence (%), Brazil, 2000–2010, both sexes, ages 5+.

State	Model 1	GGB	Model 1 minus GGB	Hybrid	Model 1 minus Hybrid
Rondônia	95	96	-1	92	4
Acre	93	94	-1	87	6
Amazonas	93	99	-6	91	2
Roraima	94	100	-6	85	9
Pará	91	80	11	77	14
Amapá	92	95	-3	84	9
Tocantins	89	96	-7	85	4
Maranhão	75	90	-15	71	4
Piauí	89	98	-9	87	2
Ceará	88	99	-11	87	0
Rio Grande do Norte	92	99	-7	89	3
Paraíba	95	98	-3	90	5
Pernambuco	97	100	-4	95	2
Alagoas	95	99	-4	92	3
Sergipe	95	101	-6	93	2
Bahia	88	98	-10	89	-1
Minas Gerais	95	100	-5	93	2
Espírito Santo	96	107	-11	99	-3
Rio de Janeiro	99	100	-2	96	3
São Paulo	98	101	-3	100	-2
Paraná	98	104	-7	99	-2
Santa Catarina	95	100	-5	94	2
Rio Grande do Sul	98	103	-4	99	-1
Mato Grosso do Sul	97	107	-10	97	0
Mato Grosso	96	100	-4	93	3
Goiás	95	99	-4	91	4
Distrito Federal	97	98	-2	101	-4
Mean absolute difference	-	-	-5	-	3
Root mean squared difference	-	-	6	-	3

GGB: Generalised Growth Balance method. Completeness for 2000–2010 for Model 1 and Model 2 was estimated by making annual estimates of completeness from 2000 to 2010, and weighting by annual completeness by the annual number of registered deaths. GGB and Hybrid estimates of completeness for both sexes were made by weighting sex-specific estimates of completeness in Queiroz et al (Tables 1 and 2) by sex-specific registered deaths [28].

## Discussion

We have proposed an empirical method to estimate the completeness of death registration that requires very few input variables and which performs well in reliably predicting completeness to guide policy action across a wide range of observed completeness, population age structures and under-five mortality rates. As expected, Model 1 outperforms Model 2 because of the inclusion of completeness of under-five mortality registration, and hence should be used where this additional information is available, and unlikely to be biased (see below). The method also predicts sex-specific completeness, which can vary substantially in some countries [18]. The models demonstrate sufficient flexibility to predict a wide range of completeness levels at a given *RegCDR*; for example, predicted completeness ranges between 22% and 99% for countries with a *RegCDR* of between 2/1000 and 3/1000. Application of the models to countries and cities not included in the sample yields predicted completeness within a few percentage points of observed completeness, with no implausible results. The models can be applied utilising data that are readily available at the subnational level to demonstrate significant differences in intra-country completeness, as the example of Colombia shows. There is strong concordance of completeness estimates from each model with those using the Hybrid DDM in Brazilian states (average difference three percentage points). However, a significant advantage of our method is that completeness can be estimated for the most recent years for which data are available (2015 for Brazil), rather than for the most recent intercensal period (2000–2010 for Brazil). Importantly, the models are intuitive and relatively straightforward, and can be incorporated into software programs to assess vital statistics data quality such as ANACONDA to predict completeness using the same age- and sex-specific death and population data inputs that are required for the program [22].

There are some limitations of the method. The overall level of mortality is represented by *5q0*, with the result that the utility of the method is diminished where the adult mortality rate is unusually high for a given *5q0*, such as in countries with high mortality from HIV/AIDS, conflict, natural disasters and alcohol-related deaths. However, this limitation is difficult to overcome given the challenges in availability and of subnational adult mortality data from surveys and censuses. The predictive strength of the models, particularly Model 1, can also be reduced where there are large uncertainty intervals around *5q0*. Sensitivity analysis shows minimal impact at the country level (a one standard error change in *5q0* resulted in a mean change in predicted completeness of 2–3 percentage points) but larger at the subnational level (mean change of 3–6 percentage points in Colombian *departmentos*). However in the absence of more reliable subnational measures of mortality, *5q0* is the most appropriate parameter to use for this method. The method also does not include other predictors likely to be associated with registration completeness, such as the resourcing of the CRVS system (e.g. population per registration offices) and socio-economic measures [32]. However, such variables were not included in the method because of challenges to accurately quantify CRVS system measures in a comparable way throughout 110 countries, as well as for other populations to correctly apply the method.

The flexibility of the method also enables it to be used to estimate the completeness of death *reporting* in a defined population, rather than just death *registration*. For example, the method could be applied to estimate the percentage of all deaths within a defined population that a health facility reports. It is recommended that Model 2 rather than Model 1 be used for such health facility data, as the completeness of under-five death reporting may be biased upwards because children are more likely to die in a health facility than people at older ages. The method is also able to estimate the completeness of reporting of household deaths in the previous 12 months derived from a census or survey.

The data used to develop the model are skewed towards country-years with high levels of completeness. The accuracy of predicted completeness is maintained at lower levels of observed completeness, but for country-years where *RegCDR* is less than 1/1000, the relative error in predicted completeness is comparatively high. However predictive precision is likely to be less of an issue for informing policy action at very low levels of completeness. Finally, the curvilinear relationship between *RegCDR* and completeness means that, for a given country, a sharp change in *RegCDR* from one year to the next may not result in a proportional change in completeness and therefore cause a material change in the implied true CDR (ie *RegCDR* divided by the completeness fraction). This would most likely be the case at lower levels of completeness where use of completeness to estimate true CDR is not recommended.

The method presented in this paper overcomes the limitations of existing methods of DDMs, capture-recapture methods and comparisons to estimates of total deaths, all of which have problems with accuracy, consistency, significant data requirements, unrealistic assumptions about population dynamics, lack of timeliness, and complexity. Rather, the method proposed here has considerable potential to be widely and easily applied by national and subnational governments to monitor the impact of efforts to improve death registration, given the importance of measuring completeness of death registration for any assessment of CRVS system performance. This will also enable countries to more confidently make use of incomplete data by adjusting them to produce reliable mortality statistics for policy.

## Supporting information

S1 Table List of countries in dataset. (PDF)

S2 Table Random effects, Model 1, both sexes. (PDF)

S3 Table Random effects, Model 2, both sexes. (PDF)

S4 Table Random effects, Model 1, males. (PDF)

S5 Table Random effects, Model 2, males. (PDF)

S6 Table Random effects, Model 1, females. (PDF)

S7 Table Random effects, Model 2, females. (PDF)

S1 Dataset Dataset used to develop models. (CSV)
