# Supplementary material for: Estimating the completeness of death registration: An empirical method
Source: PLoS One. 2018 May 30;13(5):e0197047. doi: 10.1371/journal.pone.0197047 (PMC5976169; doi:10.1371/journal.pone.0197047)
Supplement: S1 Table — (PDF) [file pone.0197047.s001.pdf]

S1 Table. List of countries in dataset

Albania	Iran	Serbia
Algeria	Iraq	Singapore
Argentina	Ireland	Slovakia
Armenia	Israel	Slovenia
Australia	Italy	Spain
Austria	Jamaica	Sri Lanka
Azerbaijan	Japan	Suriname
Bahrain	Jordan	Sweden
Barbados	Kazakhstan	Switzerland
Belarus	Kuwait	Syria
Belgium	Kyrgyzstan	Taiwan
Belize	Latvia	Tajikistan
Bolivia	Libya	Thailand
Bosnia and Herzegovina	Lithuania	The Bahamas
Brazil	Luxembourg	Trinidad and Tobago
Brunei	Macedonia	Turkey
Bulgaria	Malaysia	Turkmenistan
Canada	Maldives	Ukraine
Cape Verde	Malta	United Arab Emirates
Chile	Mauritius	United Kingdom
Colombia	Moldova	United States
Congo	Mongolia	Uruguay
Costa Rica	Montenegro	Uzbekistan
Croatia	Morocco	Venezuela
Cuba	Myanmar	
Cyprus	Netherlands	
Czech Republic	New Zealand	
Denmark	Nicaragua	
Dominican Republic	Norway	
Egypt	Oman	
El Salvador	Palestine	
Estonia	Panama	
Fiji	Papua New Guinea	
Finland	Paraguay	
France	Peru	
Georgia	Philippines	
Germany	Poland	
Greece	Portugal	
Guatemala	Puerto Rico	
Guyana	Qatar	
Honduras	Romania	
Hungary	Russia	
Iceland	Saudi Arabia	
