# Supplementary material for: Estimating the completeness of death registration: An empirical method
Source: PLoS One. 2018 May 30;13(5):e0197047. doi: 10.1371/journal.pone.0197047 (PMC5976169; doi:10.1371/journal.pone.0197047)
Supplement: S2 Table — (PDF) [file pone.0197047.s002.pdf]

S2 Table. Random effects, Model 1, both sexes

Albania	0.2000	Iran	0.2174	Serbia	-1.0113
Algeria	0.1717	Iraq	-0.1976	Singapore	0.5824
Argentina	1.0838	Ireland	0.3344	Slovakia	0.4166
Armenia	0.3338	Israel	-0.0098	Slovenia	-1.1617
Australia	0.4170	Italy	-0.0518	Spain	0.2440
Austria	0.1004	Jamaica	-0.0084	Sri Lanka	0.1461
Azerbaijan	0.1193	Japan	0.1074	Suriname	0.0170
Bahrain	-1.3729	Jordan	-0.2577	Sweden	-0.2070
Barbados	0.1618	Kazakhstan	-0.3473	Switzerland	0.5159
Belarus	0.1578	Kuwait	1.0664	Syria	-0.1140
Belgium	-0.0184	Kyrgyzstan	-0.2087	Taiwan	0.8155
Belize	-0.3732	Latvia	0.4640	Tajikistan	-0.0328
Bolivia	0.1083	Libya	-0.2326	Thailand	-0.3807
Bosnia and Herzegovina	-0.8148	Lithuania	0.1309	The Bahamas	-1.2540
Brazil	0.2966	Luxembourg	0.0259	Trinidad and Tobago	0.2029
Brunei	-0.0614	Macedonia	-1.0319	Turkey	0.5234
Bulgaria	0.0901	Malaysia	0.1249	Turkmenistan	-0.1751
Canada	1.1465	Maldives	0.1363	Ukraine	0.2073
Cape Verde	0.0743	Malta	-0.3192	United Arab Emirates	-1.5400
Chile	0.8419	Mauritius	0.6596	United Kingdom	0.2271
Colombia	-0.0739	Moldova	-1.0196	United States	0.8994
Congo	-0.2040	Mongolia	-0.7242	Uruguay	0.4178
Costa Rica	0.6970	Montenegro	-0.5715	Uzbekistan	-0.1961
Croatia	-1.5261	Morocco	0.1395	Venezuela	1.5645
Cuba	0.2810	Myanmar	-0.1599		
Cyprus	-0.7286	Netherlands	0.4879		
Czech Republic	-0.2041	New Zealand	0.3784		
Denmark	-0.0242	Nicaragua	0.2464		
Dominican Republic	0.1870	Norway	0.2624		
Egypt	0.0907	Oman	-0.9823		
El Salvador	-0.0943	Palestine	-0.0127		
Estonia	-0.1456	Panama	0.5594		
Fiji	-1.0249	Papua New Guinea	-0.1687		
Finland	-0.4415	Paraguay	0.0246		
France	0.8033	Peru	0.3055		
Georgia	-0.0369	Philippines	-0.0767		
Germany	0.3033	Poland	0.5143		
Greece	0.3059	Portugal	-0.1149		
Guatemala	0.0633	Puerto Rico	0.7522		
Guyana	-0.5396	Qatar	-0.6170		
Honduras	-0.6664	Romania	0.6079		
Hungary	-0.3753	Russia	0.5332		
Iceland	-0.2385	Saudi Arabia	-0.7437		
