# Supplementary material for: Estimating the completeness of death registration: An empirical method
Source: PLoS One. 2018 May 30;13(5):e0197047. doi: 10.1371/journal.pone.0197047 (PMC5976169; doi:10.1371/journal.pone.0197047)
Supplement: S3 Table — (PDF) [file pone.0197047.s003.pdf]

S3 Table. Random effects, Model 2, both sexes

Albania	0.0947	Iran	-0.1810	Serbia	-0.9261
Algeria	0.5955	Iraq	-0.6267	Singapore	0.6483
Argentina	1.4820	Ireland	0.3181	Slovakia	0.4400
Armenia	-0.0890	Israel	0.1413	Slovenia	-1.3128
Australia	0.5498	Italy	0.0815	Spain	0.3785
Austria	0.1444	Jamaica	-0.5700	Sri Lanka	0.4865
Azerbaijan	-0.2894	Japan	0.2633	Suriname	-0.3602
Bahrain	-1.0147	Jordan	-0.5153	Sweden	-0.2712
Barbados	0.4302	Kazakhstan	-0.5488	Switzerland	0.6031
Belarus	-0.2705	Kuwait	1.5877	Syria	-0.2949
Belgium	0.0109	Kyrgyzstan	-0.2380	Taiwan	0.9647
Belize	-0.2406	Latvia	0.4874	Tajikistan	-0.2669
Bolivia	0.1259	Libya	0.0851	Thailand	-0.9281
Bosnia and Herzegovina	-0.7420	Lithuania	0.1492	The Bahamas	-1.0747
Brazil	0.2211	Luxembourg	-0.0693	Trinidad and Tobago	-0.2343
Brunei	0.0696	Macedonia	-0.9134	Turkey	0.2221
Bulgaria	0.2281	Malaysia	0.3575	Turkmenistan	0.0057
Canada	1.3278	Maldives	0.3824	Ukraine	-0.1058
Cape Verde	0.3919	Malta	-0.2381	United Arab Emirates	-1.2749
Chile	1.2106	Mauritius	0.9305	United Kingdom	0.2698
Colombia	-0.0513	Moldova	-1.4272	United States	1.0286
Congo	-0.1038	Mongolia	-0.6796	Uruguay	0.7361
Costa Rica	1.0279	Montenegro	-0.4671	Uzbekistan	-0.1867
Croatia	-1.5776	Morocco	-0.0864	Venezuela	2.1006
Cuba	0.5251	Myanmar	-0.3804		
Cyprus	-0.7848	Netherlands	0.5337		
Czech Republic	-0.3351	New Zealand	0.5048		
Denmark	-0.0832	Nicaragua	0.3025		
Dominican Republic	-0.1237	Norway	0.2446		
Egypt	0.1771	Oman	-0.7509		
El Salvador	-0.3848	Palestine	-0.4482		
Estonia	-0.1793	Panama	0.9597		
Fiji	-1.1177	Papua New Guinea	-0.5042		
Finland	-0.5880	Paraguay	-0.0765		
France	0.9545	Peru	0.1467		
Georgia	-0.5063	Philippines	-0.3174		
Germany	0.3448	Poland	0.5923		
Greece	0.4593	Portugal	-0.0033		
Guatemala	0.3628	Puerto Rico	1.0329		
Guyana	-0.9640	Qatar	-0.5081		
Honduras	-1.0543	Romania	0.8647		
Hungary	-0.3661	Russia	0.5711		
Iceland	-0.3334	Saudi Arabia	-1.1690		
