# Supplementary material for: Estimating the completeness of death registration: An empirical method
Source: PLoS One. 2018 May 30;13(5):e0197047. doi: 10.1371/journal.pone.0197047 (PMC5976169; doi:10.1371/journal.pone.0197047)
Supplement: S4 Table — (PDF) [file pone.0197047.s004.pdf]

S4 Table. Random effects, Model 1, males

Albania	0.1570	Iraq	-0.1450	Slovakia	0.1805
Algeria	0.4780	Ireland	-0.0335	Slovenia	-0.8265
Argentina	0.8900	Israel	-0.3762	Spain	0.5046
Armenia	0.2040	Italy	0.2756	Sri Lanka	-0.1830
Australia	0.7733	Jamaica	0.2080	Suriname	0.0702
Austria	0.4858	Japan	0.3077	Sweden	0.1487
Azerbaijan	0.0168	Jordan	-0.3136	Switzerland	0.3162
Bahrain	-1.2815	Kazakhstan	-0.5496	Syria	-0.1549
Barbados	-0.3922	Kuwait	1.1673	Taiwan	0.5790
Belarus	-0.0279	Kyrgyzstan	-0.2311	Tajikistan	0.0445
Belgium	0.3462	Latvia	0.1166	Thailand	-0.4706
Belize	-0.4551	Libya	0.1100	The Bahamas	-1.1970
Bolivia	0.1495	Lithuania	0.4054	Trinidad and Tobago	0.1251
Bosnia and Herzegovina	-0.7417	Luxembourg	-0.5187	Turkey	0.3540
Brazil	0.2719	Macedonia	-1.0371	Turkmenistan	-0.1483
Brunei	-0.2693	Malaysia	0.0147	Ukraine	0.1736
Bulgaria	0.3709	Malta	-0.5082	United Arab Emirates	-1.5067
Canada	1.3417	Mauritius	0.2052	United Kingdom	0.2939
Cape Verde	-0.5196	Moldova	-0.9525	United States	1.1507
Chile	1.0393	Mongolia	-0.6402	Uruguay	0.3279
Colombia	-0.0749	Montenegro	0.0837	Uzbekistan	-0.2438
Costa Rica	0.7500	Morocco	0.4637	Venezuela	1.4527
Croatia	-1.5203	Myanmar	-0.0798		
Cuba	0.3195	Netherlands	0.6341		
Cyprus	-0.5878	New Zealand	0.2355		
Czech Republic	-0.0606	Nicaragua	0.3307		
Denmark	0.3451	Norway	0.5205		
Dominican Republic	0.1894	Oman	-0.6978		
Egypt	0.1982	Palestine	-0.0270		
El Salvador	-0.1474	Panama	0.4858		
Estonia	-0.0271	Papua New Guinea	-0.2093		
Fiji	-1.0460	Paraguay	0.1080		
Finland	-0.4913	Peru	0.3440		
France	0.2385	Philippines	-0.0975		
Georgia	-0.4013	Poland	0.7964		
Germany	0.3429	Portugal	0.0181		
Greece	-0.0562	Puerto Rico	-0.0370		
Guatemala	0.4118	Qatar	-0.6030		
Guyana	-0.5924	Romania	0.5581		
Honduras	-0.4895	Russia	0.8658		
Hungary	-0.2866	Saudi Arabia	-0.4669		
Iceland	-0.8633	Serbia	-1.0104		
Iran	0.1364	Singapore	0.1646		
