# Supplementary material for: Estimating the completeness of death registration: An empirical method
Source: PLoS One. 2018 May 30;13(5):e0197047. doi: 10.1371/journal.pone.0197047 (PMC5976169; doi:10.1371/journal.pone.0197047)
Supplement: S5 Table — (PDF) [file pone.0197047.s005.pdf]

S5 Table. Random effects, Model 2, males

Albania	0.0417	Iraq	-0.5210	Slovakia	0.2042
Algeria	0.9400	Ireland	-0.0342	Slovenia	-0.9779
Argentina	1.2187	Israel	-0.2263	Spain	0.5858
Armenia	-0.2048	Italy	0.3591	Sri Lanka	0.1092
Australia	0.8942	Jamaica	-0.2595	Suriname	-0.2715
Austria	0.5206	Japan	0.3716	Sweden	0.0994
Azerbaijan	-0.3572	Jordan	-0.5463	Switzerland	0.3727
Bahrain	-0.8876	Kazakhstan	-0.7553	Syria	-0.3077
Barbados	-0.1386	Kuwait	1.6742	Taiwan	0.6925
Belarus	-0.4490	Kyrgyzstan	-0.2469	Tajikistan	-0.1328
Belgium	0.3713	Latvia	0.0869	Thailand	-0.9784
Belize	-0.2999	Libya	0.4544	The Bahamas	-1.0203
Bolivia	0.1651	Lithuania	0.3966	Trinidad and Tobago	-0.2529
Bosnia and Herzegovina	-0.6461	Luxembourg	-0.6339	Turkey	0.0313
Brazil	0.1852	Macedonia	-0.8892	Turkmenistan	0.0427
Brunei	-0.1646	Malaysia	0.2540	Ukraine	-0.1543
Bulgaria	0.5394	Malta	-0.4010	United Arab Emirates	-1.1605
Canada	1.4899	Mauritius	0.4427	United Kingdom	0.3437
Cape Verde	-0.2341	Moldova	-1.2877	United States	1.2448
Chile	1.3688	Mongolia	-0.5564	Uruguay	0.5863
Colombia	-0.0747	Montenegro	0.2578	Uzbekistan	-0.2255
Costa Rica	1.0560	Morocco	0.1963	Venezuela	1.9169
Croatia	-1.5827	Myanmar	-0.3049		
Cuba	0.5580	Netherlands	0.6861		
Cyprus	-0.5889	New Zealand	0.3478		
Czech Republic	-0.1803	Nicaragua	0.3823		
Denmark	0.3320	Norway	0.5138		
Dominican Republic	-0.0846	Oman	-0.3999		
Egypt	0.2989	Palestine	-0.3928		
El Salvador	-0.4360	Panama	0.8399		
Estonia	-0.0985	Papua New Guinea	-0.5313		
Fiji	-1.0901	Paraguay	0.0513		
Finland	-0.6617	Peru	0.2186		
France	0.3117	Philippines	-0.3083		
Georgia	-0.8756	Poland	0.8579		
Germany	0.3584	Portugal	0.0910		
Greece	0.0387	Puerto Rico	0.1083		
Guatemala	0.7173	Qatar	-0.4264		
Guyana	-0.9451	Romania	0.8011		
Honduras	-0.8080	Russia	0.8760		
Hungary	-0.2635	Saudi Arabia	-0.8046		
Iceland	-0.9390	Serbia	-0.8889		
Iran	-0.2088	Singapore	0.2131		
