# Supplementary material for: Estimating the completeness of death registration: An empirical method
Source: PLoS One. 2018 May 30;13(5):e0197047. doi: 10.1371/journal.pone.0197047 (PMC5976169; doi:10.1371/journal.pone.0197047)
Supplement: S6 Table — (PDF) [file pone.0197047.s006.pdf]

S6 Table. Random effects, Model 1, females

Albania	0.3585	Ireland	-0.0803	Slovakia	0.5133
Algeria	0.0473	Israel	0.1373	Slovenia	-1.0807
Argentina	0.7192	Italy	0.1950	Spain	0.3409
Armenia	0.6396	Jamaica	-0.2551	Sri Lanka	0.9982
Australia	0.4297	Japan	0.3899	Suriname	0.0446
Austria	0.1247	Jordan	-0.5191	Sweden	-0.1613
Azerbaijan	0.2745	Kazakhstan	-0.1139	Switzerland	0.3416
Bahrain	-1.4168	Kuwait	0.6684	Syria	-0.0759
Barbados	-0.1693	Kyrgyzstan	-0.0117	Taiwan	0.6906
Belarus	0.5854	Latvia	0.4024	Tajikistan	-0.1154
Belgium	-0.0373	Libya	-0.5064	Thailand	-0.2841
Belize	-0.2275	Lithuania	-0.1122	The Bahamas	-1.2689
Bolivia	0.0419	Luxembourg	-0.4862	Trinidad and Tobago	0.7493
Bosnia and Herzegovina	-0.7243	Macedonia	-0.8222	Turkey	0.7250
Brazil	0.4513	Malaysia	0.3321	Turkmenistan	-0.0679
Bulgaria	0.3175	Maldives	-0.2575	Ukraine	0.6511
Canada	0.9255	Malta	-0.4095	United Arab Emirates	-1.4188
Cape Verde	0.6302	Mauritius	0.5314	United Kingdom	0.1332
Chile	0.6820	Moldova	-0.9159	United States	1.0710
Colombia	-0.0050	Mongolia	-0.6790	Uruguay	0.6588
Costa Rica	0.6881	Montenegro	-0.8182	Uzbekistan	-0.3991
Croatia	-1.1031	Morocco	-0.0732	Venezuela	1.7568
Cuba	0.2212	Myanmar	-0.1452		
Cyprus	-1.1047	Netherlands	0.3750		
Czech Republic	-0.2166	New Zealand	0.1828		
Denmark	-0.2802	Nicaragua	0.1586		
Dominican Republic	0.1206	Norway	0.1516		
Egypt	0.1291	Oman	-1.2523		
El Salvador	0.0276	Palestine	-0.1556		
Estonia	0.1282	Panama	0.6239		
Fiji	-0.8543	Papua New Guinea	-0.2987		
Finland	-0.5019	Paraguay	-0.0898		
France	0.6269	Peru	0.2411		
Georgia	0.4297	Philippines	-0.0088		
Germany	0.3284	Poland	0.3883		
Greece	0.4460	Portugal	0.0553		
Guatemala	0.0689	Puerto Rico	0.7616		
Guyana	-0.4746	Qatar	-0.3299		
Honduras	-0.9435	Romania	0.5298		
Hungary	-0.1819	Russia	0.5728		
Iceland	-0.7510	Saudi Arabia	-1.3151		
Iran	0.1810	Serbia	-1.1828		
Iraq	-0.3538	Singapore	0.0617		
