# Supplementary material for: Estimating the completeness of death registration: An empirical method
Source: PLoS One. 2018 May 30;13(5):e0197047. doi: 10.1371/journal.pone.0197047 (PMC5976169; doi:10.1371/journal.pone.0197047)
Supplement: S7 Table — (PDF) [file pone.0197047.s007.pdf]

S7 Table. Random effects, Model 2, females

Albania	0.2838	Ireland	-0.1321	Slovakia	0.5529
Algeria	0.4291	Israel	0.2435	Slovenia	-1.1943
Argentina	1.0777	Italy	0.3113	Spain	0.4635
Armenia	0.2739	Jamaica	-0.8098	Sri Lanka	1.3715
Australia	0.5237	Japan	0.5598	Suriname	-0.2735
Austria	0.1293	Jordan	-0.7187	Sweden	-0.2607
Azerbaijan	-0.0574	Kazakhstan	-0.2326	Switzerland	0.3837
Bahrain	-1.1079	Kuwait	1.1050	Syria	-0.2406
Barbados	0.0176	Kyrgyzstan	-0.0029	Taiwan	0.8554
Belarus	0.2419	Latvia	0.4253	Tajikistan	-0.3325
Belgium	-0.0394	Libya	-0.2186	Thailand	-0.7361
Belize	-0.0567	Lithuania	-0.0567	The Bahamas	-1.0565
Bolivia	0.0333	Luxembourg	-0.6127	Trinidad and Tobago	0.3682
Bosnia and Herzegovina	-0.6482	Macedonia	-0.7071	Turkey	0.4702
Brazil	0.4268	Malaysia	0.5557	Turkmenistan	0.1156
Bulgaria	0.4249	Maldives	-0.1490	Ukraine	0.3978
Canada	1.0659	Malta	-0.4024	United Arab Emirates	-1.1476
Cape Verde	1.0385	Mauritius	0.7894	United Kingdom	0.1229
Chile	1.0129	Moldova	-1.2689	United States	1.1798
Colombia	0.0625	Mongolia	-0.6014	Uruguay	0.9698
Costa Rica	0.9678	Montenegro	-0.7465	Uzbekistan	-0.4026
Croatia	-1.1105	Morocco	-0.2037	Venezuela	2.2453
Cuba	0.4160	Myanmar	-0.3800		
Cyprus	-1.2030	Netherlands	0.3808		
Czech Republic	-0.3461	New Zealand	0.2581		
Denmark	-0.3754	Nicaragua	0.2098		
Dominican Republic	-0.1677	Norway	0.0968		
Egypt	0.2239	Oman	-1.0219		
El Salvador	-0.1710	Palestine	-0.5624		
Estonia	0.1440	Panama	0.9851		
Fiji	-0.9038	Papua New Guinea	-0.6527		
Finland	-0.6142	Paraguay	-0.2143		
France	0.7365	Peru	0.0734		
Georgia	0.0332	Philippines	-0.1849		
Germany	0.3320	Poland	0.4824		
Greece	0.5706	Portugal	0.1550		
Guatemala	0.3657	Puerto Rico	1.0508		
Guyana	-0.8232	Qatar	-0.2355		
Honduras	-1.3083	Romania	0.7424		
Hungary	-0.1538	Russia	0.6572		
Iceland	-0.8882	Saudi Arabia	-1.7123		
Iran	-0.1730	Serbia	-1.1238		
Iraq	-0.7732	Singapore	0.1061		
